# Elevated temperature during rearing diminishes swimming and disturbs the metabolism of yellow perch larvae

**DOI:** 10.1242/jeb.250164

**Published:** 2025-10-23

**Authors:** Mellissa Easwaramoorthy, William Andrew Thompson, Shamaila Fraz, Joshua P. Nederveen, Peyton Hartenstein, Lisa Laframboise, Richard G. Manzon, Christopher M. Somers, Joanna Y. Wilson

**Affiliations:** ^1^ Department of Biology, McMaster University, 1280 Main St West, Hamilton, ON, Canada, L8S 4K1; ^2^ Department of Kinesiology, McMaster University, 1280 Main St West, Hamilton, ON, Canada, L8S 4K1; ^3^ Department of Pediatrics, Faculty of Health Sciences, McMaster University Medical Center, 1200 Main St W, Hamilton, ON, Canada, L8N 3Z5; ^4^ Department of Biology, University of Regina, 3737 Wascana Parkway, Regina, SK, Canada, S4S 0A2

**Keywords:** Thermal effluent, Climate change, Native species, Metabolic dysfunction

## Abstract

Temperate waters, such as the Great Lakes, are predicted to increase in temperature by 1°C every decade. Many poikilothermic fish thermoregulate behaviourally, moving to more suitable thermal environments. Embryos are incapable of locomotion and may be exposed to non-optimal temperatures during development. Previous work has suggested that temperature increases during embryogenesis can alter growth rates in fish. However, less is known of whether these early-life exposures to elevated temperatures can impart alterations to the phenotypic plasticity of performance traits, particularly in temperate species. We hypothesized that increased embryonic incubation temperature would diminish the larval performance of yellow perch (*Perca flavescens*), a critical cultural and ecological species of fish. We reared yellow perch embryos at 12, 15 or 18°C until hatching; after hatching, the temperature was raised to a common garden 18°C, their preferred post-hatch temperature. We assessed exploratory behaviour, metabolism (oxygen consumption) and cardiac performance throughout early development. At hatch, 12°C fish exhibited the greatest swimming activity, with 18°C fish consuming the least oxygen and possibly experiencing mitochondrial dysfunction. Cardiac development was more advanced at hatch in 18°C fish. Yet, warmer incubated fish had diminished movement and increased oxygen consumption at 20 days post-hatch, demonstrating long-term disruptions of increased temperature in the embryonic environment. Overall, elevations in rearing temperature may cause metabolic dysfunction and behavioural alterations, potentially impacting the survival of yellow perch.

## INTRODUCTION

Anthropogenic activities and subsequent climate change are warming surface water temperatures of the Great Lakes of North America, which are expected to increase by as much as 6°C by 2071–2100 (Trumpickas et al., 2009). Increased water temperature could prove detrimental to ectothermic aquatic species that depend on their environment to determine internal body temperature, influencing numerous basic physiological processes such as metabolism and cardiovascular function (Rozen-Rechels et al., 2019). Aquatic species are particularly vulnerable to warming water temperatures in the embryonic period, as immotile embryos are incapable of behaviourally thermoregulating by moving to more tolerable thermal environments (Cowan et al., 2023). Fish embryos may cope with sub-optimal environmental temperature during early critical windows by altering the development and function of major physiological systems, although these developmental alterations could be deleterious to longer-term survival.

Embryonic metabolic rates increase with elevations in temperature, in part driven by increases in oxygen consumption rates (Eme et al., 2015). Adaptive responses are necessary to maintain homeostasis, matching metabolic demands to the prevailing oxygen availability (Nakazawa et al., 2016). However, there is often a mismatch between the heightened demand for oxygen and the capacity of the fish cardiorespiratory system to transport this oxygen at increasing temperatures (Jensen et al., 2017). For example, the inability of cardiometabolic systems to meet the amplified demands for oxygen in sockeye salmon (*Oncorhynchus nerka*) at supra-optimal temperatures led to cardiorespiratory collapse during exhaustive exercise (Burnett et al., 2014). This problem is exaggerated in embryos as their ability to take up oxygen and other nutrients is limited by their surface area to volume ratio (Burggren, 2004), so a balance is maintained between growth and metabolic rates (Martin et al., 2020). Warmer waters also have reduced dissolved oxygen concentrations, exacerbating oxygen limitations (Walczyńska and Sobczyk, 2017). Studies investigating the relationship between elevated embryonic temperatures and growth profiles indicate that rainbow trout (*Oncorhynchus mykiss*), lake whitefish (*Coregonus clupeaformi*s) and common minnow (*Phoxinus phoxinus*) embryos experience rapid developmental rates and a shorter time to hatch (Melendez and Mueller, 2021; Mueller et al., 2015; Schönweger et al., 2000). These changes result in smaller fish at hatch with decreased length, yolk-free dry mass and elevated mortality rates (Eme et al., 2015; Melendez and Mueller, 2021; Mueller et al., 2015).

Apart from immediate changes to physiology, the temperature experienced throughout embryogenesis can impart long-term changes to phenotypic plasticity in fish (Koumoundouros et al., 2009; Kourkouta et al., 2021; Scott and Johnston, 2012). Increased temperatures during embryonic windows can lead to reductions in critical swimming speed in European sea bass (*Dicentrarchus labrax*; Koumoundouros et al., 2009) juveniles and gilthead seabream (*Sparus aurata*; Kourkouta et al., 2021) larvae. Conversely, elevations in embryonic temperature led to improved swimming speeds following thermal acclimation (Scott and Johnston, 2012). Together, this suggests that the long-term effects of elevated temperatures during embryogenesis may be species and life-stage specific. However, the long-term effects of warming temperatures during embryogenesis on cold or cool-water freshwater species are largely unknown.

This study tested the hypothesis that elevated embryonic incubation temperatures could impart long-term impacts on the phenotypic plasticity of a cool-water fish, the yellow perch. The yellow perch is a species native to a large proportion of North America, and significant decreases in population sizes have been observed recently by Indigenous communities (Rancourt, 2017). Elevated incubation temperature alters perch developmental rate, survival to hatching and the incidence of developmental abnormalities (Fraz et al., 2024). Changes in the underlying cardiometabolic function that dictate the growth and development of these animals have yet to be explored. Beyond individual physiological alterations, measures of their behavioural activity may also shed light on whole-animal performance, which is particularly important for foraging, obtaining shelter and evading predators during the vulnerable post-hatch period (Plaut, 2001). To investigate the effect of temperature on rearing, yellow perch embryos were incubated at 12, 15 and 18°C. These temperatures were chosen to match existing and predicted spring water temperatures in the Great Lakes region due to the effects of global warming in the coming decades (Trumpickas et al., 2009). Post-hatch, the optimal temperature of this species increases (Hokanson and Kleiner, 1974); hence, water temperatures were gradually increased to a common temperature of 18°C. In some studies, incubation temperatures of 15°C maximize the survival and growth of yellow perch (Hokanson and Kleiner, 1974) while in other perch studies, 15°C incubation is associated with lower survival compared with both lower and higher incubation temperatures (Fraz et al., 2024). Environmental temperature is typically lower (6–12°C) at spawn (Hart et al., 2006) and increases during embryogenesis; thus, 12 and 15°C are more optimal for embryogenesis and 18°C incubation is considered non-optimal. The experimental test points were chosen based on a prior study demonstrating the critical developmental stages of yellow perch when reared in an experimental design similar to this study (Fraz et al., 2024, 2025). The hatching stage to 20 days post-hatch (dph) is the period during which all yellow perch larvae, regardless of early embryonic temperature, transition from yolk sac absorption to exogenous feeding by mouth (Fraz et al., 2024). As a result, we expect their physiological development and behaviour to mirror this in order to survive this key transition. We characterized cardiac development by assessing markers at hatch and 20 dph. Whole-animal metabolic rates were measured at hatch and 5, 10 and 20 dph. To probe into possible changes in mitochondrial respiration, we estimated basal metabolic rate, ATP production, proton leak and non-mitochondrial respiration. To gauge locomotory function in larvae, we performed general swimming assessments from hatch until 5 dph, and at 10 and 20 dph. At hatch, transcript abundance was determined for vascular endothelial growth factor-A (*vegfa*), myosin heavy chain (*myhc*) and NKX2-homeobox 5 (*nkx2.5*), genes important for blood vasculature (Schuermann et al., 2014; Burggren, 2004), muscle (Schiaffino et al., 2015) and cardiac development (Harrington et al., 2017), respectively. Apoptotic cell death (hatch) and the circulatory system (pre-hatch and at hatch) were determined in whole animals. The results of this study suggest that elevated embryonic incubation temperature can cause long-term impacts on the performance of yellow perch but may not be directly related to perturbations seen in cardiometabolic function.

## MATERIALS AND METHODS

### Fish source and embryo incubation

Sexually mature adult yellow perch, *Perca flavescens* (Mitchill 1814), females (*n*=20) and males (*n*=10) were acquired by angling from Mitchell's Bay on Lake St Clair, ON, Canada (42.47°N, 82.41°W) on 26 March and 2 April 2023, and held in naturalized ponds (natural spring temperature and day–night cycle; live minnow feed; Leadley Environmental Corp, Essex, ON, Canada). The water temperature at the site of collection and in the naturalized ponds was not recorded. Fish were transported in two coolers (filled with 90 l of pond water; 15 fish per cooler) and received aeration, which maintained the temperature from the time of collection (12°C) until arrival at McMaster University (Hamilton, ON, Canada; 3 h transport) on 11 April 2023. Fish were acclimated in dechlorinated city tap water with spawning substrate and fed frozen chironomid larvae twice a day (150 l holding tank; Omega One Freeze-Dried Bloodworms, Big Al's Canada) for 1 week (12–13°C; 12 h:12 h day–night cycle). Fish were held in groups of 15, with a 2:1 female-to-male ratio to induce egg spawning; females were anaesthetized (MS-222; 80 mg l^−1^ and 160 mg l^−1^ sodium bicarbonate in dechlorinated city tap water) and injected with human chorionic gonadotropin (1 µl g^−1^ at 300 IU kg^−1^ in 0.9% saline), allowed to recover in an aerated 30 l container, and then left undisturbed in new holding tanks under holding conditions (3 fish; 2:1 female:male ratio). A second injection was performed 3 days later, following this procedure, as this process led to a near-complete induction of spawning for injected females. Typically, the third morning following the second injection, egg ribbons were collected, weighed and checked for fertilization. Viable ribbons were cut transversely to flatten the ribbon and cut into six equal sections. Two of these sections were used in each of the temperature groups to create two replicates of the study. A total of 12 ribbons were used to collect the data presented in this study, for a total of 24 replicates. Ribbons were placed into approximately 2 l of E_2_ embryo media (15 mmol l^−1^ NaCl; 0.5 mmol l^−1^ KCl; 1 mmol l^−1^ MgSO_4_; 0.15 mmol l^−1^ KH_2_PO_4_; 0.05 mmol l^−1^ Na_2_HPO_4_; 1 mmol l^−1^ CaCl_2_; 0.7 mmol l^−1^ NaHCO_3_) and placed into incubators at 12 and 15°C. For the 18°C condition, ribbons were allowed to acclimate to 15°C for 2 h before transfer to 18°C.

**Fig. 1.**
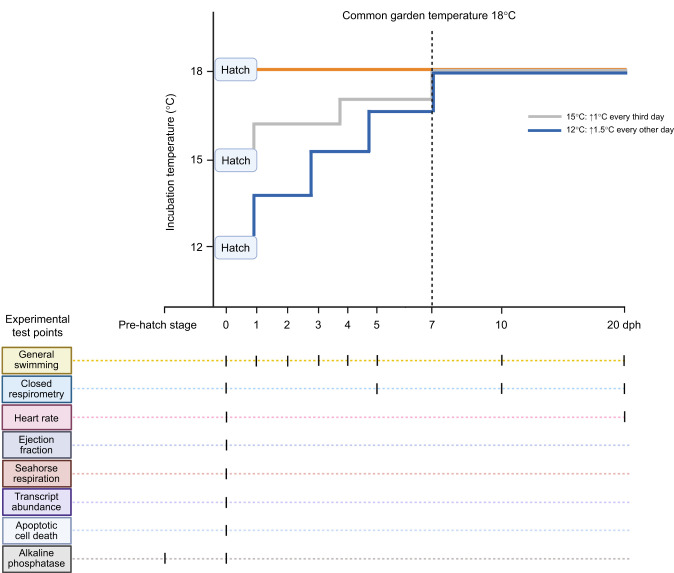
**Experimental design and test points.** The illustration shows the ramp-up design of 12, 15 and 18°C incubation temperature groups from hatch to 20 days post-hatch (dph). The test points at which each experiment was run are shown as tick marks.

From 48 h post-fertilization until the eye pigmentation stage, ribbons were treated with 0.01% neutral buffered formalin every second day to reduce fungal growth, and water was changed daily. At hatch, incubation temperatures were increased every third day for 15°C (+1°C), and every other day for 12°C (+1.5°C) groups, until all treatments were at a common garden condition of 18°C (by 7 dph). After hatching, fish began feed training with a combination of Gemma^®^ micro 300 (Skretting, Salt Lake City, UT, USA) and first instar brine shrimp; a light source was placed above tanks during feeding to encourage perch to surface feed. Routine animal care was conducted by L.L., W.A.T., M.E., S.F. and P.H. Adult yellow perch collection and spawning protocols were approved and in accordance with McMaster University's animal research ethics board (AUP# 20–06-23). A schematic diagram of the temperature exposure and sampling can be found in Fig. 1.

### Animal sampling

Embryos were sampled by W.A.T. at the onset of heartbeat, eye pigmentation and hatch. Yellow perch were euthanized by overdose exposure to MS-222 (400 mg l^−1^ and 800 mg l^−1^ sodium bicarbonate in embryo media). For alkaline phosphatase and Acridine Orange staining (see Supplementary Materials and Methods), whole embryos or larvae were fixed in 10% neutral buffered formalin overnight at 4°C. The following day, samples were transferred to 70% ethanol and stored at 4°C until quantification. For quantitative PCR (qPCR), 12–15 larvae at hatch were pooled from one replicate, across eight replicates, flash-frozen in liquid nitrogen and stored at −80°C.

### Behavioural assay

Yellow perch larvae were assessed for exploratory behaviour by W.A.T. and M.E. using a general swimming assay, tracking movement immediately after introduction to a novel environment. Individual fish shortly after hatch and at 1, 2, 3, 4, 5 and 10 dph were transferred into a 24-well clear plate in 2 ml per well E2 media (1 individual per well). Fish at 20 dph were transferred to a 12-well clear plate with 5 ml per well E2 media to account for the change in overall growth between 10 and 20 dph (Fraz et al., 2024). Plates were moved to the Daniovision observation chamber (Noldus, Wageningen, The Netherlands) and recorded for 20 min under natural light. The total distance travelled and the maximum velocity were determined using Ethovision XT software (version 15.0.1416; Noldus). Individuals that did not move were removed from further statistical analysis (total *n*=42–48).

### Closed respirometry

**Fig. 2.**
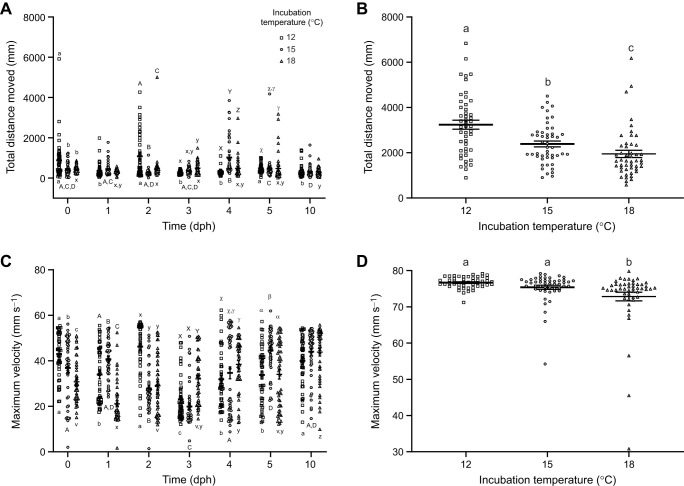
**Elevated incubation temperature reduces behavioural performance in post-hatch *Perca flavescens* larvae.** The total distance travelled of yellow perch larvae incubated at 12, 15 and 18°C at (A) hatch to 10 dph and (B) 20 dph, and the maximum velocity of larvae at (C) hatch to 10 dph and (D) 20 dph, from 20 min observations of larvae at hatch and 1, 2, 3, 4, 5, 10 and 20 dph (means±s.e.m.; individual data points overlayed). At hatch and 1, 2, 3, 4, 5, 10 and 20 dph, *n*=44, 46, 47, 47, 48, 48, 48, 48 for the 12°C group; *n*=42, 48, 48, 48, 48, 48, 48, 48 for the 15°C group; *n*=48, 48, 48, 48, 48, 48, 48, 48 for the 18°C group, respectively. Different letters denote significant differences between incubation temperatures (above dataset; a/b/c; A/B/C/D; v/x/y; X/Y/Z; γ/χ; α/β) or developmental age (below dataset; a/b/c; A/B/C/D; v/x/y/z), and are not comparable across other factors (developmental age or incubation temperature, respectively).

M.E. conducted closed respirometry to determine oxygen uptake rate as a measure of metabolic rate in the animal. This was measured at the respective incubation temperatures of fish at hatch and 5, 10 and 20 dph from each treatment group (12, 15 and 18°C). Additional closed respirometry trials were run by S.F., assisted by P.H. These trials were at hatch at the respective incubation temperatures and at 25°C. The higher matched that at which the Agilent Seahorse mitochondrial assay were collected, to confirm animal viability. The replicates for oxygen consumption analyses ranged from 12 to 18 per group; specific replicates per age and treatment are given in the Fig. 2 caption and Table S1.

Four oxygen respiration vials (4 ml volume, 15 mm diameter, 48 mm height) with integrated oxygen sensors were connected to the Pyro Science FireSting-O2 (four channels; Aachen, Germany) containing data logging software. Respirometry was conducted at noon in standard room lighting conditions. The oxygen sensors were calibrated before each use with air-saturated water at each respective incubation temperature on the day of assessment. To maintain appropriate fish mass to water volume ratios (g ml^−1^) within the respirometer, a 1:100 ratio of larvae was added to three of the four respirometers (approximately 20, 15, 10 and 5 larvae at hatch and 5 10 and 20 dph, respectively). One respirometer vial in every trial contained only E2 water to account for background microbial respiration. Respirometers were submerged in an insulated and temperature-regulated water bath, with a circulating pump to ensure mixing. Oxygen saturation values were collected from individual vials every 5 s for 1 h. Immediately after, the pool of larvae in each vial was weighed. Oxygen saturation values were analysed by M.E. with guidance from W.A.T. in R (version 4.3.0; http://www.R-project.org/) and RStudio (version 2023.06.0+421; https://posit.co/download/rstudio-desktop/) using the ‘Respirometry’ package. The first 15 min contained noisy oxygen saturation values due to the initial handling of the larvae; therefore, this period was trimmed from our analysis. Data were analysed in 20 min bins to allow for the highest *R*^2^ values. Therefore, three slopes were measured: 15–35, 20–40 and 25–45 min, like a rolling regression model (Harianto et al., 2019). Oxygen consumption rates (OCRs) derived from these slopes had to satisfy two criteria to be included in further analysis: *R*^2^ value >0.9 and a higher limit of oxygen saturation levels ≥70%. This ensured that low, more variable data and periods of potentially hypoxic conditions were considered outliers. After averaging slope values that passed these criteria, blanks (background respiration) were subtracted. To account for variation in body mass, measurements were normalized by larval mass; data normalized by individual fish are provided in Table S1. An error in the mass collection during the acute exposure to 25°C prevented us from correcting oxygen consumption to be mass specific for these samples.

### Seahorse mitochondrial respiration assay

The Seahorse mitochondrial respiration assay was performed by J.P.N. Mitochondrial respiration was measured by the Seahorse XFe 24 Extracellular Flux Analyzer (Agilent, Santa Clara, CA, USA), using XF24 Islet Capture Microplates. Yellow perch were collected on the day of hatch and incubated at 25°C for 1 h prior to the assay. The Seahorse XF Calibrant fluid was used to hydrate the sensor plate for 24 h at 25°C before the assay. To evaluate OCR, 94 μmol l^−1^ of oligomycin (Sigma-Aldrich), 29 μmol l^−1^ of carbonyl cyanide-*p*-trifluoromethoxyphenylhydrazone (FCCP; Sigma-Aldrich) and 219 μmol l^−1^ of rotenone and antimycin A (Sigma-Aldrich) were added in the injecting assay media, in sequence. One fish from each temperature group was placed into an individual well, and background wells (*n*=4) contained no fish. The Seahorse XFe24 Bioanalyzer was scheduled to complete a cycle of a mix for 2 min, followed by a waiting period of 1 min, and measure for 2 min within each measurement cycle. Initial baseline data were collected over 5 cycles, followed by the injection of oligomycin. Basal respiration levels were calculated as the baseline OCR prior to the injection of oligomycin. Oligomycin, an inhibitor of ATP synthase, results in a decrease in OCR once injected. This decrease represents the amount of oxygen utilized in respiration coupled with ATP production, another metric that was calculated. Following the injection of oligomycin, OCR was measured from 5 cycles before the injection of FCCP; 5 cycles were measured following the injection of FCCP. The final injection was a combination of rotenone and antimycin A, after which the last 5 cycles were measured. Non-mitochondrial respiration was determined as the difference between the very last OCR after the addition of rotenone/antimycin A, a cocktail of inhibitors of cellular respiration, and zero. Proton leak results in the incomplete coupling of substrate oxidative phosphorylation and ATP production, and was calculated from the difference between the remaining basal respiration levels after the addition of oligomycin and non-mitochondrial respiration. While we did use FCCP in this assay, the uncoupler was not capable of raising respiration higher than basal respiration levels, and this result was not considered (*n*=14–16).

### Heart rate and ejection fraction

Yellow perch were maintained at the appropriate incubation temperature using a temperature-regulated water bath. Videos of the heart were approximately 30 s and recorded by M.E. and W.A.T. on an Axio Zoom V16 microscope (Carl Zeiss, Oberkochen, Germany) connected to a Canon EOS Rebel T1i camera using AxioVision software (Rel. 4.8; Carl Zeiss). At hatch, videos were recorded at the incubation temperature and after an acute increase to 18°C (rate increased 3°C 30 min^−1^). Yellow perch at 20 dph were recorded at 18°C only because they were all at the same common garden temperature; individual larvae were immobilized using mild anaesthesia for 2 min before recording. Using VLC media player (v.3.0; Paris, France), videos were slowed to 0.25–0.5 times and trimmed (20–30 s). Heartbeats were visually counted by M.E. a total of 3 times per video with the observer blind to the treatment and converted to beats min^−1^. At hatch (*n*=37–50), fish were recorded at their respective incubation temperatures and then raised acutely to 18°C, and heart rate was recorded for 12 and 15°C groups again (*n*=44–45). Heart rate for each group at 20 dph was recorded at 18°C (*n*=30–31).

Ejection fraction was estimated by M.E. from the videos, using the image analysis protocol of Perrichon et al. (2017). Suitable clips were chosen, videos were examined frame by frame, and screenshots were captured at the precise moment of end-diastole (maximum ventricle volume) and end-systole (minimum ventricle volume) for 5 continuous cardiac cycles. Using ImageJ (Fiji; v.1.54f), the perimeter of the cardiac ventricle was traced with the freehand ROI tool and parameters were set to include the best-fitted ellipse within the selected area. From the best-fitted ellipse, measurements of the major (*a*) and minor (*b*) axes were estimated by ImageJ and input into the volume of a prolate spheroid equation to quantify ventricle volume:
(1)

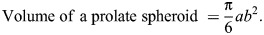
Once diastolic and systolic volumes were calculated for each fish at hatch, the difference between the two measures was considered the stroke volume. However, to account for differences in zoom magnification (no standardized scale was used), ejection fraction was calculated and expressed as relative changes in volume (percentage difference) per cycle. This was repeated for each of the 5 cycles of diastolic and systolic events per larvae and the ejection fraction (%) was averaged (*n*=21–29):
(2)

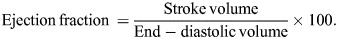


### qPCR

Quantification of transcript abundance was carried out by M.E. under guidance from W.A.T. as described by Thompson and Vijayan (2020). RNA was extracted from a total of 8 samples containing 12–15 pooled fish using TRIzol reagent as per manufacturer’s instructions (cat. no. 15596018, Thermo-Fisher Scientific, Waltham, MA, USA) with the addition of glycogen to enhance precipitation (RNA grade; cat. no. r0551, Thermo-Fisher Scientific). A total of 1 μg of RNA was used to create cDNA using a QuantiTect Reverse Transcription Kit (cat. no. 205311, Qiagen, Hilden, Germany). Before qPCR, testing was conducted for optimal annealing temperature via a primer PCR gradient (Taq DNA Polymerase cat. no. 10342053, Thermo-Fisher Scientific). The optimal annealing temperature was 60°C for all primers (Table S2). Our primers were for the genes vascular endothelial growth factor- A (*vegfa*), myosin heavy chain (*myhc*) and NKX2- homeobox 5 (*nkx2.5*) and were confirmed via sequencing after amplification in yellow perch samples. qPCR was performed with SYBR Green Supermix (cat. no. 1725270, Bio-Rad, Hercules, CA, USA) using a CFX96 Real-Time System C1000 Touch thermal cycler (Bio-Rad). The raw Cq (quantification cycle) values were obtained using CFX Manager Software (v.3.1, Bio-Rad). The Monte Carlo Markov Chain algorithm (MCMC.qPCR package; version 0.9-7; https://CRAN.R-project.org/package=MCMC.qpcr) was used to compare the relative abundance of mRNA transcripts without the use of reference genes. The MCMC algorithm samples from the joint posterior distribution of all model parameters to estimate the effects of experimental factors on the process of expression amplification (Matz et al., 2013). qPCR results are expressed as log_2_ gene abundance values, which are the posterior means with 95% credible intervals (Matz et al., 2013). These posterior mean values were interpreted as significantly different when the credible intervals did not overlap. This analysis was conducted with five genes; data from three genes are included in this publication.

### Statistical analysis

W.A.T. and M.E. conducted all statistical analyses in this manuscript using R (version 4.3.0) and RStudio (version 2023.06.0+421). When the assumptions of a normal distribution or equal variance were not met, transformations were performed; otherwise, a non-parametric test was employed. Untransformed data are shown throughout this paper. Tests were conducted using a Kruskal–Wallis one-way ANOVA followed by a *post hoc* Dunn test, a one-way ANOVA followed by a Tukey honest significant difference test (HSD), or a two-way ANOVA followed by a Tukey HSD test. As we used two different arena sizes for our behavioural assessment, and arena size can directly influence behaviour (Padilla et al., 2011; Näslund et al., 2015), fish at 20 dph were analysed separately.

## RESULTS

### Behaviour

There was a significant interaction between developmental age and temperature (*F*_12,975_=12.53, *P*<0.0001; two-way ANOVA; Fig. 2A) for the distance fish moved. At hatch, 12°C fish moved significantly more than fish reared at warmer temperatures, a result that was also seen at 2 dph (*P*<0.05). However, at 3 dph, 18°C fish moved more than 12°C fish (*P*<0.05). At 4 dph, 15°C fish moved the most, with 18°C fish also moving more than 12°C fish; at 5 dph, 12°C fish moved more than 18°C fish (*P*<0.05). Developmentally, 12°C fish moved less distance at 1, 3, 4 and 10 dph, compared with movement observed at hatch (*P*<0.05). A peak in movement was observed at 4 dph in 15°C fish (*P*<0.05), while movement dropped in fish reared at 18°C at 10 dph relative to hatch (*P*<0.05). At 20 dph, there was a significant effect of incubation temperature on distance travelled (*P*<0.0001; Kruskal–Wallis; Fig. 2B), with 12°C fish moving the most, and 18°C fish moving the least (*P*<0.05).

The maximum velocity also had a significant interaction effect (*F*_12,975_=18.92, *P*<0.0001; two-way ANOVA; Fig. 2C). At hatch, maximum velocity was significantly different between all groups, with the velocity of 12°C fish being the highest and that of 18°C fish being the lowest (*P*<0.05). All the groups exhibited a different maximum velocity at 1 dph, with 15°C fish having the highest velocity, followed by 12°C fish, and 18°C fish having the lowest (*P*<0.05). Maximum velocity was higher in 12°C fish at 2 dph, which were significantly greater than that of 15 and 18°C fish (*P*<0.05). At 3 dph, 18°C fish had a significantly higher maximum velocity relative to colder conspecifics (*P*<0.05). The 18°C fish maintained a higher maximum velocity at 4 dph, which was significantly elevated relative to that of the 12°C fish (*P*<0.05). At 5 dph, 15°C fish had the highest maximum velocity when compared with warmer and colder fish (*P*<0.05). There were no differences in maximum velocity among groups at 10 dph (*P*>0.05). Developmentally, 12°C fish exhibit a lowered maximum velocity at 1, 3, 4 and 5 dph relative to hatch (*P*<0.05). Similarly, 15°C larvae at 2 and 3 dph had a lowered velocity compared with hatch, with increased maximum velocities after 5 dph (*P*<0.05). The 18°C fish had a dip in their maximum velocity at 1 dph relative to hatch, and an increase in maximum velocity at 3, 4 and 10 dph (*P*<0.05). Maximum velocity was also affected at 20 dph (*P*<0.0001; Kruskal–Wallis; Fig. 2D), with both 12 and 15°C fish exhibiting a higher maximum velocity than 18°C fish (*P*<0.05).

### OCR

**Fig. 3.**
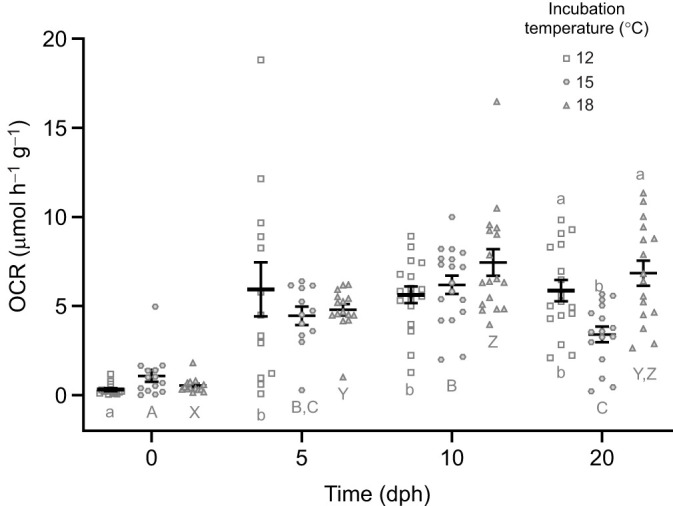
**Incubation temperature influences oxygen consumption post-hatch.** Oxygen consumption rates are shown for yellow perch larvae that were incubated at 12, 15 and 18°C at hatch and 5, 10 and 20 dph. At hatch and 5, 10 and 20 dph, *n*=14, 13, 18, 17 for the 12°C group; *n*=14, 12, 18, 16 for the 15°C group: *n*=14, 15, 17, 16 for the 18°C group, respectively. Different letters are used to denote significant differences between incubation temperatures (above dataset; a/b) or developmental age (below dataset; a/b; A/B/C; X/Y/Z), and are not comparable across other factors (developmental age or incubation temperature, respectively).

There was a significant interaction between temperature and developmental age in regard to OCR (*F*_6,172_=3.08, *P*<0.0069; two-way ANOVA; Fig. 3). There was an effect of incubation temperature on OCR (µmol h^−1^ g^−1^) at 20 dph, with 15°C fish having a lower rate than 12 and 18°C fish (*P*<0.01). Developmentally, the oxygen consumption of 12°C fish increased at 5 dph, remaining elevated at 10 and 20 dph (*P*<0.05). A similar result was seen with 15°C fish, with a higher OCR at 5, 10 and 20 dph relative to that at hatch (*P*<0.05). The 18°C fish also had a higher OCR at 5, 10 and 20 dph compared with hatch (*P*<0.05).

OCR normalized for individual larvae (μmol h^−1^ larvae^−1^) showed no significant differences (*P*>0.05; Kruskal–Wallis; Table S1). Acute increases in temperature to 25°C (to match Seahorse mitochondrial respiration assay measurements) revealed significant impacts on OCR (*F*_2,64_=4.7, *P*=0.013; two-way ANOVA; Fig. S1), with fish incubated at 12°C having a higher metabolic rate than 18°C fish (*P*=0.009). Yellow perch were confirmed viable with the acute increase in temperature at hatch.

### Seahorse mitochondrial respiration assay

**Fig. 4.**
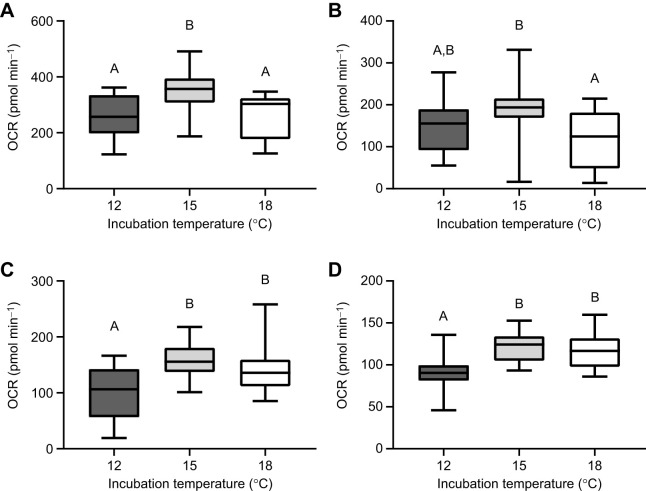
**Elevated incubation temperatures may impose bioenergetic dysfunction.** The OCR associated with (A) basal respiration, (B) ATP-linked respiration, (C) proton leak and (D) non-mitochondrial respiration at hatch in yellow perch incubated at 12, 15 or 18°C. *n*=14, 16, 16 for 12, 15 and 18°C larvae, respectively. Different letters are used to denote significant differences between groups. For box plots, lower and upper boundaries of the box represent the 25th and 75th percentiles of the data, the line in the centre of the box represents the median, and error bars encompass the entirety of the data spread.

Measures of basal respiration were significantly higher in yellow perch at hatch (*F*_2,43_=6.82, *P*=0.003; one-way ANOVA; Fig. 4A) from the 15°C incubation temperature relative to 12°C (*P*<0.007) and 18°C (*P*=0.007) fish. ATP-linked respiration was higher in yellow perch at hatch incubated at 15°C (*F*_2,43_=4.04, *P*=0.025; one-way ANOVA; Fig. 4B) compared with that in those incubated at 18°C (*P*<0.021). The OCR associated with proton leak significantly increased with incubation temperature in yellow perch (*F*_2,43_=6.93, *P*<0.003; one-way ANOVA; Fig. 4C). Fish incubated at 12°C exhibited a lower OCR regarding proton leak compared with those incubated at 15°C (*P*=0.0023) and 18°C (*P*=0.023). Non-mitochondrial respiration also increased with incubation temperature in yellow perch (*F*_2,43_=10.1, *P*=0.0003; one-way ANOVA; Fig. 4D). At hatch, fish incubated at both 15 and 18°C increased OCR for non-mitochondrial respiration measurements when compared with 12°C fish (*P*=0.0005 and *P*<0.002, respectively).

### Heart rate and ejection fraction

**Fig. 5.**
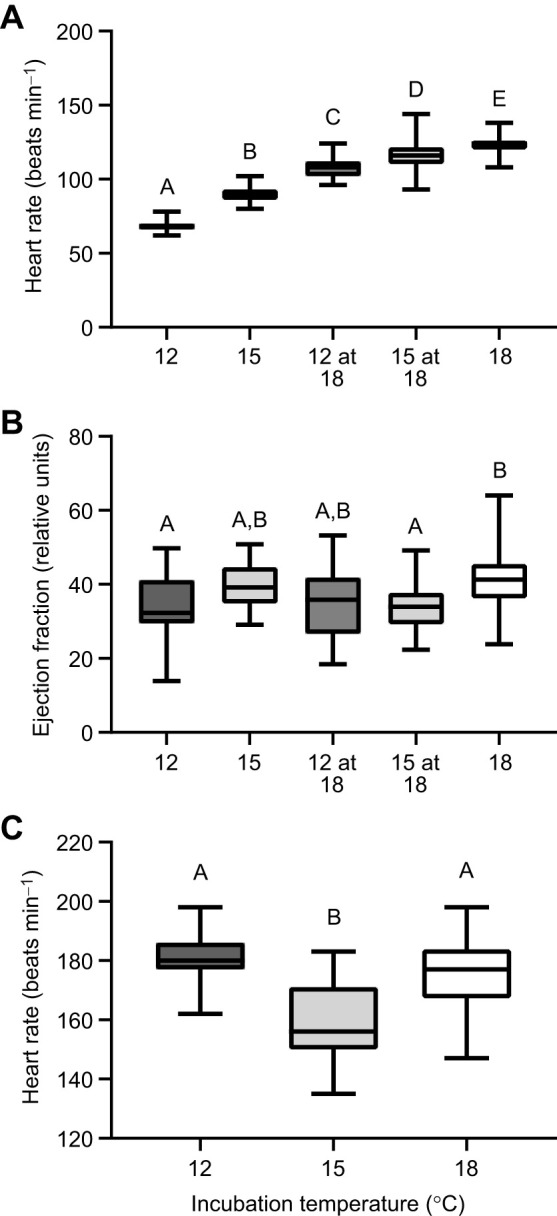
**Cardiac performance is affected by incubation temperature.** (A) Heart rate and (B) ejection fraction of yellow perch larvae at hatch at either their ambient temperature (12 or 15°C) or 18°C. (C) Heart rate of 20 dph larvae (all held at 18°C). At ambient hatch temperatures, *n*=50, 48, 37 for heart rate of 12, 15 and 18°C larvae; *n*=45, 44 for 12 and 15°C groups at 18°C (A). At ambient hatch temperatures, *n*=21, 29, 24 for ejection fraction of 12, 15 and 18°C groups; *n*=22, 27 for 12 and 15°C groups at 18°C, respectively (B). At 20 dph, *n*=30, 31, 30 for 12, 15 and 18°C groups, respectively (C). Different letters denote significant differences between groups. For box plots, lower and upper boundaries of the box represent the 25th and 75th percentiles of the data, the line in the centre of the box represents the median, and error bars encompass the entirety of the data spread.

Incubation temperature had a significant effect on heart rate at hatch (*F*_4,201_=589.7; *P*<0.0001; one-way ANOVA; Fig. 5A). Heart rate increased with temperature, with 12°C fish possessing the lowest heart rate, and 18°C fish having the highest heart rate (*P*<0.05). Correcting for ambient temperature, and raising 12 and 15°C fish to 18°C did increase heart rate relative to their holding conditions (12 and 15°C, respectively), but heart rate was still lower than that of fish incubated at 18°C (*P*<0.05). At 20 dph, incubation temperature altered heart rate (*F*_2,88_=27.02, *P*<0.0001; one-way ANOVA; Fig. 5C) and was lowest in 15°C fish compared with 18°C fish (*P*<0.0001; one-way ANOVA) and 12°C fish (*P*<0.0001; one-way ANOVA). There was no significant difference (*P*>0.05; one-way ANOVA) in heart rate between 12 and 18°C fish at 20 dph.

Incubation temperature had a significant effect (*F*_4,118_=4.226 *P*=0.0031; one-way ANOVA; Fig. 5B) on the ejection fraction of fish at hatch. Fish incubated at 12°C had a lower ejection fraction than those at 18°C (*P*=0.012) but those incubated at 15°C were not different (*P*>0.05) from either. However, when the heart was observed at a common temperature (acute increase to 18°C), the ejection fraction was lower for fish incubated at 15°C than for those incubated at 18°C (*P*=0.014).

### Cardiovascular transcript abundance

**Fig. 6.**
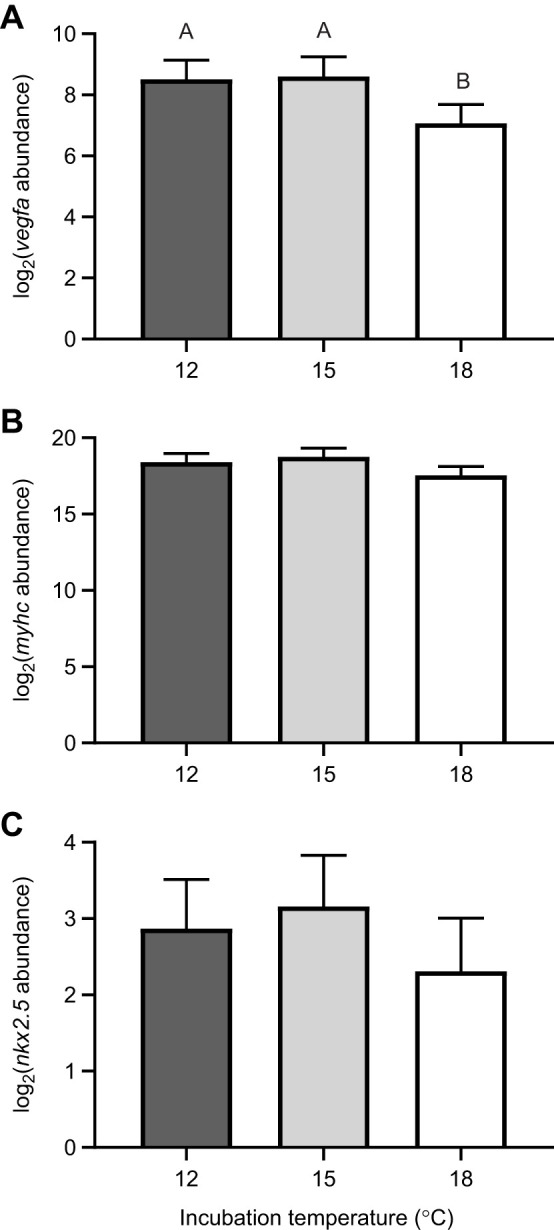
**Higher incubation temperature reduces the transcript abundance of *vegfa*.** Relative gene abundance of (A) *vegfa*, (B) *myhc* and (C) *nkx2.5* in yellow perch incubated at 12, 15 and 18°C at hatch. *n*=16 for all groups. Different letters represent significant differences between groups. Data are means, with error bars denoting 95% credible intervals.

The expression of *vegfa* was significantly lower in the 18°C treatment compared with the 12°C (*P*=0.021; MCMC) and 15°C (*P*=0.017; MCMC) treatments at hatch (Fig. 6A). The expression of *myhc* and *nkx2.5* was not different (*P*>0.05) between fish incubated at 12, 15 and 18°C at hatch (Fig. 6B,C).

### Apoptosis and vasculature staining

At hatch, incubation temperatures significantly affected (*H*_2_=56.14, *P*<0.0001; Kruskal–Wallis; Fig. S2B) whole-body apoptotic cell death. Fish incubated at 15°C had lower apoptosis compared with those incubated at 12°C (*P*<0.0001) and 18°C (*P*< 0.0001). The 12°C group had the highest whole-body fluorescence readings, higher than those at 18°C (*P*<0.003). Within the heart, there was no effect (*P*>0.05; one-way ANOVA; Fig. S2A). There were no changes in vascularization in the heart, brain or muscle across incubation temperatures for any time point measured (Figs S3 and S4).

## DISCUSSION

This study provides evidence that early incubation temperature can programme longer-term changes in yellow perch cardiac development, oxygen consumption and performance. While previous studies have demonstrated that increased temperatures during embryogenesis could lead to changes in growth plasticity post-hatch in yellow perch (Fraz et al., 2024, 2025), no study has assessed long-term changes in metabolism or animal performance in this species. Here, we present evidence that elevations in incubation temperature impose metabolic dysfunction and disturbed behavioural swimming profiles at hatch, possibly indicative of perturbed ecological performance at a critical life stage. A behavioural advantage of higher activity was noted at 20 dph in fish incubated at 12°C, while fish incubated in warmer temperatures (15 and 18°C) were hypoactive by comparison. The warmer temperatures used in this study may be ecologically relevant based on current freshwater projections from climate change models (Trumpickas et al., 2009). Together, these results lend credence to the idea that warmer incubations may be detrimental to the post-hatch development and subsequent survival of the yellow perch.

The capacity of fish to maximize swimming performance is considered a critical determining factor in their ability to survive (Plaut, 2001). Early limitations in behavioural responses may negatively impact the ability of larvae to find suitable settlement sites in aquatic habitats (Kingsford et al., 2002; Fiksen et al., 2007). To characterize early behavioural responses in the yellow perch, we utilized a high-resolution assessment of early swimming behaviour, observing significant declines in the performance of fish incubated in warmer waters. General swimming assays offer an estimation of the possible foraging, migration and predator avoidance of fish (Plaut, 2001), and insight into the maintenance activity of undisturbed animals (Fisher and Leis, 2010). Increased distances travelled, for instance, may reflect the total search capacity an animal would undertake in a novel environment, or its general energetic expenditure (Letcher et al., 1996). Conversely, the maximum velocity, or the top speed exhibited by the animal during the assessment, may be representative of swimming ability in larval fish (Leis et al., 2007). We demonstrated that at hatch, fish incubated in colder waters (12°C) typically travel a greater distance and have a higher maximum velocity compared with their warmer conspecifics, suggesting that colder fish may have advanced swimming capacity immediately following hatch. Given that there were no differences in *myhc* gene expression, a major contractile protein in fast skeletal muscle (Nord et al., 2014), differences in skeletal muscle abundance may not be different across groups. Interestingly, metabolic rates and swim performance are also not well aligned. Metabolism, particularly maximal respiration, is more closely linked to maximal assessments of behaviour, either via a swim tunnel or following aggressive acts (Metcalfe et al., 2015). More expansive characterizations of behavioural performance during the early hatching window may be prudent to understand whether these alterations in exploratory behaviour are indicative of maximal larval performance.

We originally hypothesized that changes in metabolism could explain the differences in swim performance with incubation temperatures. However, the metabolic rates we obtained at hatch were surprising, and are likely to be a reflection of a combination of homeostatic maintenance processes (Rolfe and Brown, 1997), growth, non-mitochondrial oxygen consumption and activity (Sokolova, 2021). Temperature increases can lead to a concomitant increase in metabolic rate (Eme et al., 2015; Melendez and Mueller, 2021), and we predicted a higher metabolic rate in fish incubated in warmer waters. Instead, we only observed a significant elevation in oxygen consumption in 15°C fish compared with 18°C fish in our Seahorse mitochondrial respiration assay measurements. Behaviourally, colder fish also appear to move more throughout early development, which may indicate that metabolic rate differences between these incubation temperatures result from the effects of temperature on non-activity-based energy sinks. Although we observed a slight change in ATP-linked OCRs in 12°C fish relative to their basal respiration, overall, we describe a parallel pattern between respiration and ATP-linked OCRs across incubation groups, suggesting that the control of ATP synthase over respiration does not change with incubation temperature. Instead, at the whole-animal level, warmer fish are perhaps experiencing metabolic dysfunction.

The evidence for possible disruption to metabolism stems from estimations of proton leak and non-mitochondrial respiration. Proton leak reflects a mechanism of regulating ATP synthesis, while reducing the production of harmful reactive oxygen species (ROS) (Rolfe and Brand, 1997), and comprises a significant amount of the total respiration of animal cells (Sokolova, 2021). Non-mitochondrial respiration, representing mechanisms of oxygen consumption not coupled to proton pumping or ATP production, is composed of oxidase reactions (such as peroxisomal fatty acid oxidation) and collectively has been suggested to consume 10% of the whole-body oxygen of rats (Rolfe and Brown, 1997). Warmer fish appear to show increases in proton leak, while also increasing non-mitochondrial respiration. This is important, as increases in proton leak have been seen with elevations in temperature (Mueller et al., 2011; Onukwufor et al., 2015), raising the cost of energy required to maintain mitochondrial integrity. This, in turn, may suggest an increased need to elevate respiration to counteract the increased cost of oxidative phosphorylation. Indeed, while 15°C fish showed elevations in non-mitochondrial respiration, they also displayed increases in total respiration. In contrast, in the 18°C group, an elevation in non-mitochondrial OCR did not occur in parallel with an increase in total oxygen consumption, possibly suggestive of impairment in mitochondrial ATP production. One caveat with our measurements in the Seahorse Bioanalyzer is that the data had to be collected at 25°C. Simultaneous measurements of oxygen uptake at this temperature and their incubation temperature revealed no significant change as a result of the acute temperature increase, indicating a limited impact of increased temperature (Fig. S1). Future studies investigating mitochondrial plasticity following temperature increases in this species are necessary to understand the potential impacts of climate change.

Our results suggest that the heart may play a limited role in performance at hatch. Cardiometabolic systems govern the ability of the body to pump blood from the heart and effectively deliver oxygenated blood to be used for ATP production in energy-demanding tissues. In vertebrates such as fish, the heart is the first organ to develop. Fish begin to depend on their cardiovascular system when transcutaneous diffusion of oxygen and nutrients can no longer support the high rate of embryonic growth (Burggren, 2004). The early-life exposure of numerous fish species such as the lake whitefish (*Coregonus clupeaformis*; Lim et al., 2017), mahi mahi (*Coryphaena hippurus*; Perrichon et al., 2017), common minnow (*Phoxinus phoxinus*; Schönweger et al., 2000) and zebrafish (*Danio rerio*; Barrionuevo and Burggren, 1999) embryos to elevated incubation temperatures results in significantly higher heart rates throughout development, which may be suggestive of functional disruptions to the heart following perturbed cardiac development (Perrichon et al., 2017). In this study, fish exposed to higher incubation temperatures exhibited higher heart rates and ejection fractions at their ambient incubation temperatures at hatch. Suspecting a possible direct temperature effect on the heart, we tested whether the patterns remained at a common temperature (18°C) and found that even when fish were measured at a common temperature, those incubated at an elevated temperature possessed a higher heart rate and ejection fraction. Our measurements of *nkx2.5* transcript expression at hatch, a cardiac developmental marker (Burggren, 2004), were similar across all temperature groups, suggesting that the yellow perch heart may be developmentally equivalent at hatch across all groups. If we assume development is the same, this then allows us to propose that there is a functional change in the early heart as a result of the incubation temperature. For example, both 12 and 15°C groups at hatch elevate their heart rate when acutely raised to 18°C, but only the 12°C fish can maintain the volume of blood pumped with each heartbeat. Atlantic salmon (*Salmo salar*) presented lower ventricular mass and a lower percentage of spongy myocardium, thereby decreasing the overall force production responsible for blood ejection (Muir et al., 2022). Yet, it is important to consider that in the study by Muir et al. (2022), cardiac output, and stroke volume in particular, did not vary with incubation temperature, unlike for the yellow perch in this study. The overt consequence of these varied cardiac responses of yellow perch is unknown, as metabolically we do not see a link between oxygen consumption and heart rate at hatch. While a relationship has been well established in adult fish between cardiac performance and oxygen consumption (Webber et al., 1998; Schönweger et al., 2000), there is likely no link between these measures at early embryonic and larval periods in the yellow perch. It has been clearly demonstrated that oxygen consumption at the early larval period may be via transcutaneous diffusion of oxygen and independent of the circulatory system (Burggren, 2013; Mirkovic and Rombough, 1998; Schönweger et al., 2000). It is then possible that the yellow perch utilizes the prosynchronotropy model (Burggren, 2013), where the heart starts to beat before the convective flow of oxygen and nutrients is required by the animal. Altogether, the differences in cardiac performance at hatch across incubation temperatures may be indicative of underlying changes in functional capabilities of the heart and not directly associated with oxygen consumption, but this remains to be explicitly tested.

At 5 and 10 dph, OCR was similar in all treatment groups but was increased nearly 10-fold compared with values recorded at hatch. Increases in OCR after hatch have been observed during early windows of body mass accumulation in zebrafish larvae (Barrionuevo and Burggren, 1999). At 20 dph, differences began to appear in metabolism across incubation temperatures, with both 12 and 18°C groups presenting higher heart rates and OCRs when compared with those of groups incubated at 15°C. In zebrafish, OCR increases substantially at 10 days post-fertilization but begins to significantly diminish as the animal grows (Barrionuevo and Burggren, 1999), and perhaps 15°C fish are simply reducing metabolic rate with age. It is also possible that differences in swimming activity can account for differences in OCR seen at 20 dph. For example, we note a graded change in total distance travelled at this time point, with 12°C fish moving significantly more than 15°C fish, which moved significantly more than 18°C fish. The interplay between energetic investment in movement versus growth may influence the changes in OCR observed in the present study. Fish in the 18°C treatment transition to complete dependence on exogenous sources of food between 10 and 20 dph, while 15 and 12°C-incubated larvae have been noted to take upwards of 20 days to fully rely on food by mouth (Fraz et al., 2024), further supported by a stark increase in protein observed during this window (Fraz et al., 2025). Moreover, post-hatch, 18°C fish exhibit higher growth rates relative to their colder conspecifics (Fraz et al., 2025). Perhaps the metabolic rate of 18°C-incubated fish is a by-product of the earlier onset of exogenous feeding and increased growth in this group, as feeding can elevate metabolic rates for extended durations (Leeuwan et al., 2011; Ferreira et al., 2019).

The markers of cardiovascularization investigated in this study were not predominantly affected by alterations in incubation temperature and hence may have minimal effect on the diminished swimming observed in warmer temperatures. The formation of an extensive vascular network is crucial to allow the transport of respiratory gases and nutrients to every tissue in the body (Burggren, 2004). Zebrafish studies show that the overall form of vasculature is developed before the initiation of blood circulation in fish and is reproducible between embryos (Schuermann et al., 2014). The final pattern of blood vessels is, however, highly plastic, and easily remodelled by factors in the environment during early development (Schuermann et al., 2014). Cardiac output typically increases alongside the development and growth of animals yet may be further heightened at higher incubation temperatures, causing a significant increase in blood pressure within the system (Perrichon et al., 2017). To decrease cardiovascular blood pressure via a decrease in total peripheral resistance, blood vessel formation may be upregulated by endothelial cells that secrete VEGF and trigger sprouting angiogenesis (Burggren, 2004). We observed that at hatch, yellow perch incubated at the warmest temperature had a lower transcript abundance of *vegfa* compared with those at cooler incubation temperatures. This was an interesting discovery, considering that at this time point, 18°C fish also possessed the highest heart rate, even when corrected for temperature. Suspecting changes in vascularization, we performed alkaline phosphatase staining, a method that has been shown to stain the vasculature of zebrafish (Eliceiri et al., 2011). From this assessment, we noted no significant differences across incubation temperatures from the onset of the heartbeat in the yellow perch. Given that evidence has suggested that VEGF may also act as an anti-apoptotic factor (Meeson et al., 1999), and high temperatures can induce excess ROS production, potentially leading to cellular and tissue damage via apoptosis, we used Acridine Orange stain to assess cellular death. Our whole-body measurements indicate that apoptosis was highest at 12°C, followed by 18°C and lowest at 15°C, yet these differences were not different in the heart nor were they related to *vegfa* transcript levels. The increases in whole-body apoptosis observed at 12°C may be associated with a more rapid rate of growth in the embryonic period (Fig. S2B; Fraz et al., 2024, 2025), as a mechanism to regulate cell growth (Elmore, 2007). However, reductions in *vegfa* may indicate reduced function, as recent studies using morpholino knockdowns of *vegf* have shown reductions of this transcript impair hypoxia performance in zebrafish (Hughes and Perry, 2021). Episodic hypoxic events in freshwater lakes can occur through storm-driven upwelling, severe rainfall runoffs and direct nutrient inputs from high population centres, and increasing temperatures in lake systems may lead to reductions in the mixing potential of large lakes (Tellier et al., 2022). The reductions in possible ATP production potential observed in this study may be suggestive of a particular sensitivity to withstand conditions of reduced oxygen, but will require further investigation.

### Conclusions

The early rearing environment represents a critical developmental window for an animal, programming long-term function and performance. In the Great Lakes, projections have forecast increases of nearly 0.5–1°C per decade (Hayhoe et al., 2010); however, the consequences of these projected increases in temperature on aquatic species residing in the Great Lakes are largely unknown. Considering the inability of the embryo to move to more favourable conditions and the sensitivity of this life stage to temperature, studies on the effects of elevated incubation temperatures are especially important. The temperate yellow perch has experienced population declines in recent decades, with studies proposing a link to increasing temperatures globally (Collingsworth et al., 2017; Farmer et al., 2015). This study has demonstrated that environmentally relevant 3°C increases in temperature during rearing can produce poorer swimming at hatch. Moreover, we have identified a period of development where locomotion becomes more pronounced (>10 dph) and shown that the temperature experienced during embryogenesis can dictate subsequent behavioural profiles at this time point. These changes in behaviour may be influenced by altered growth patterns, as previous studies have shown that the incubation temperature of rearing can change the developmental rate and body shape (Fraz et al., 2025). Overall, however, the poorer swimming, increased oxygen consumption and metabolic dysfunction found in fish from 18°C incubation may limit the recruitment of yellow perch and ultimately the survival of larvae. Future studies exploring the performance of these animals in terms of their responses to environmental stimuli (noise and light), and other secondary stressors, such as contaminants, is paramount for risk management of this critically important native species.

## Supplementary Material

10.1242/jexbio.250164_sup1 Supplementary information
